# Design principles governing chemomechanical coupling of kinesin

**DOI:** 10.1038/s41598-017-01328-9

**Published:** 2017-04-25

**Authors:** Tomonari Sumi

**Affiliations:** 1 Research Institute for Interdisciplinary Science, Okayama University, 3-1-1 Tsushima-Naka, Kita-ku, Okayama, 700-8530 Japan; 2 Department of Chemistry, Faculty of Science, Okayama University, 3-1-1 Tsushima-Naka, Kita-ku, Okayama, 700-8530 Japan

## Abstract

A systematic chemomechanical network model for the molecular motor kinesin is presented in this report. The network model is based on the nucleotide-dependent binding affinity of the heads to an microtubule (MT) and the asymmetries and similarities between the chemical transitions caused by the intramolecular strain between the front and rear heads. The network model allows for multiple chemomechanical cycles and takes into account all possible mechanical transitions between states in which one head is strongly bound and the other head is weakly bound to an MT. The results obtained from the model show the ATP-concentration dependence of the dominant forward stepping cycle and support a gated rear head mechanism in which the forward step is controlled by ATP hydrolysis and the resulting ADP-bound state of the rear head when the ATP level is saturated. When the ATP level is saturated, the energy from ATP hydrolysis is used to concentrate the chemical transition flux to a force-generating state that can produce the power stroke. In contrast, when the ATP level is low, the hydrolysis energy is consumed to avoid states in which the leading head is weakly bound to an MT and to inhibit frequent backward steps upon loading.

## Introduction

Kinesin-1, herein referred to simply as kinesin, is a molecular motor that transports intracellular cargo along microtubules (MTs) toward their plus ends by converting the free energy derived from the hydrolysis of ATP into mechanical work^1–7^. Kinesin’s motility is remarkably processive and proceeds by alternately advancing its two heads in a walking-like or hand-over-hand manner^8–10^. Each motor step comprises an 8-nm displacement^11^ that corresponds to the distance between adjacent *α*/*β*-tubulin dimers along an MT. When the external load is low, the motor steps are always directed toward the plus end of the MT^12, 13^, and the ratio of the number of ATP hydrolysis to the number of steps advanced suggests a tight coupling between the two^14–16^.

The remarkable processivity of kinesin and the coordination between its two heads can be explained by a gating mechanism, where a critical transition on the chemomechanical cycle of one head is inhibited until a certain specific transition proceeds in the partner head. Two general classes of gating mechanisms have been proposed, one for gating mechanical transitions and the other for gating chemical transitions. As for the former, it has been proposed that a mechanical step transition of the ADP-bound rear head is gated by ATP binding to the leading head^17, 18^. This gating mechanism is the one occurring when the ATP concentration is low, in which case the ATP binding in the leading head is expected to be rate-limiting. Another mechanism proposed for mechanical gating is that the mechanical release of the trailing head from the MT is accelerated by intramolecular strain^19^. With respect to chemical gating, it has been proposed that ATP binding in the nucleotide-free leading head is repressed until the ADP-binding rear head dissociates from the MT^20, 21^; ADP release from the rear head is inhibited until the rear head advances to a forward position, due to ATP binding in the leading head, and binds to the next tubulin-binding site^22, 23^. These gating mechanisms are based on the intramolecular strain between the two heads. In fact, the intramolecular strain affects the binding affinity of each head to the MT^24, 25^ and the binding and unbinding rates of the nucleotides in each head^26^ because the leading and trailing heads are pulled toward the backward and forward directions, respectively, via the neck linkers.

Stochastic modeling has been applied to study the chemical and mechanical properties of molecular motors^27–39^. Specifically, Liepelt and Lipowsky developed a chemomechanical network theory for two-headed molecular motors such as kinesin and myosin V^33, 35, 37, 39^. By employing such a network representation for the chemical and mechanical transitions, one can systematically construct a stochastic model of the molecular motor which leads to the numerical results that are in agreement with experimentally determined external load and nucleotide-concentration dependences of molecular motor kinetics. The advantage of this approach is that one can reduce the number of independent chemical transition rates using steady-state balance conditions^33, 34^, which are generalizations of the detailed balanced conditions at equilibrium.

In this study, we employed chemomechanical network theory to gain insight into the design principles of kinesin motors. Some key questions related to the mechanism of kinesin were summarized in a presentation by Steven Block^40^:

1. Where in the chemical transition pathway are forward steps generated?
2. What kind of gating mechanism is needed to achieve kinesin’s processive movement?
3. When kinesin is sped up by an assisting force, is it going through its normal chemical transition cycle or via some other pathway?
4. Is the backstepping cycle a reversal of the forward cycle, and does kinesin generate ATP under superstall loads that force it to move backward?

To shed light on these questions, we applied the network approach to kinesin and systematically compared the model results with available single-molecule observation data for the external load dependence on the motor velocity, ratio of forward to backward steps, mean run length, and detachment rate from MT at various ATP concentrations. Importantly, we did not impose the restriction of a pre-selected working cycle, as was done in earlier theoretical works^27–29, 31^, nor did we impose a restriction to a specific mechanical transition as in Liepelt’s and Lipowsky’s works^33, 35, 37^. Instead, based on both the nucleotide-dependent binding affinity of the head for an MT and the effects of the intramolecular strain between two heads on the chemical transitions, we newly introduced asymmetries and similarity relationships between the chemical transition rates to reduce the number of unknowns systematically. Furthermore, by taking into account all mechanical transitions between states in which one head is strongly bound to the MT and the other head is weakly bound to the MT, the network model can provide the motor properties that are in good agreement with the force-generating directed motility caused by ATP hydrolysis and the ATP-free bidirectional passive movements assisted by an external force, as observed experimentally, using only a set of the chemical and mechanical transition rates. We conclude this report with an explanation of how ATP hydrolysis energy is consumed by kinesin to attain the observed extraordinary motor properties from the chemomechanical network point of view.

## Chemomechanical network modeling of kinesin

### Kinesin’s state space

**Figure 1:**
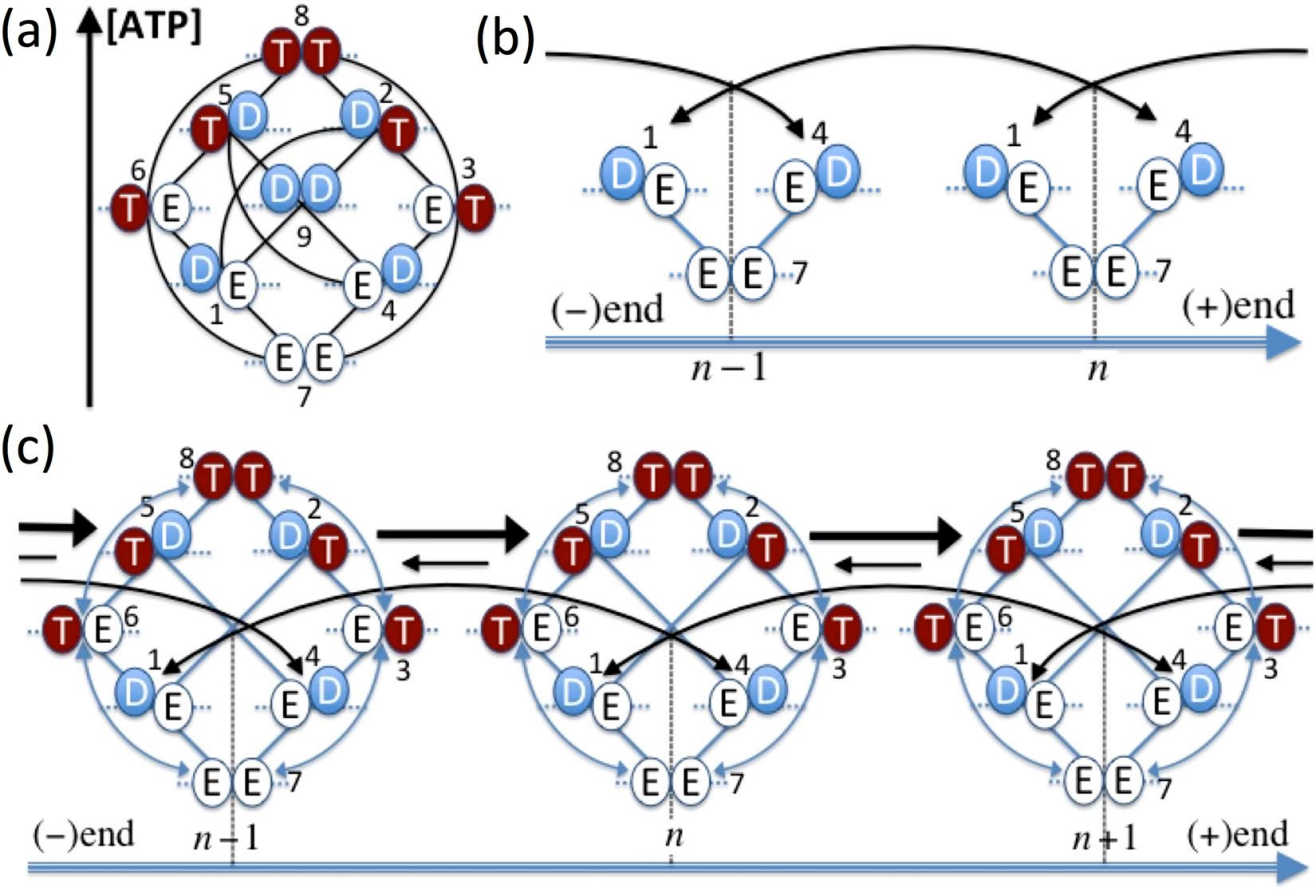
(**a**) Kinesin state space consisting of 3^2^ = 9 chemical states and 18 chemical transitions between these states^33^, (**b**) a three-state model for ATP-free force-assisted passive bidirectional movement of kinesin at finite ADP concentrations, and (**c**) an eight-state chemomechanical network model of kinesin. In these figures, kinesin heads with ATP and ADP bound are denoted by T and D, respectively, and empty heads are denoted by E. Both the T and E states are strongly bound to an MT, whereas the D state is weakly bound^25, 41, 42^. The vertical displacements of the heads with the D state from the baseline indicate their weak binding. In Fig. 1a, the left axis schematically indicates that the appearance probability of each state qualitatively depends on the ATP concentration. In the absence of ATP (Fig. 1b), kinesin’s movement is always a symmetric random walk irrespective of ADP concentration, as long as no external force is applied. In Fig. 1c, the weakest binding state, state 9 (DD), contributes neither a mechanical step transition nor supports staying on an MT, so it is eliminated from the full state space shown in Fig. 1a, while all possible chemical and mechanical transitions among the other eight states are taken into account. Detachment of kinesin from the MT is assumed to occur via states 2 (DT), 5 (TD), 1 (DE), and 4 (ED) that are related to the mechanical step (see “Calculation details” of the Method section and Supplementary Information).

To describe the chemomechanical dynamics of the kinesin motor, we applied the chemomechanical network theory presented by Liepelt and Lipowsky^33^. In this approach, the catalytic cycle of an ATPase with a single catalytic domain is modeled using three chemical states, those in which the catalytic domain is empty (E), occupied by ATP (T), and occupied by ADP (D). Thus, the catalytic cycle of kinesin, a two-headed molecular motor with one catalytic domain per head, can be expressed using a network representation with 3^2^ = 9 different chemical states, as shown in Fig. 1a. The network representation based on the nine-state space provided a starting point for our systematic modeling of the complex chemomechanical coupling of the two-headed motors. The binding affinity of kinesin’s head for MT has been demonstrated to depend on the chemical state of the head^25, 41, 42^. A head in state E or state T is strongly bound to an MT, while a head in state D is weakly bound to an MT. The nucleotide-state-dependent binding affinity of each head is indicated as the vertical displacement of each head from the dotted baseline in Fig. 1.

### Mechanical step transitions

If kinesin walks via a hand-over-hand manner^8–10^, the leading and trailing heads exchange their positions on an MT during mechanical step transitions. To make a mechanical step, one head should be strongly bound to an MT while the other head should be weakly bound. In the full nine-state network representation [Fig. 1a], this requirement for the mechanical step leads to two mechanical transitions between states 2 (DT) and 5 (TD) and between states 1 (DE) and 4 (ED). Force-generating processive movements of kinesin are well known never to be observed in the absence of ATP, even if ADP is sufficiently supplied. This observation suggests that the mechanical transition from state 2 (DT) to state 5 (TD), which includes an ATP-binding head, should be the main contributor to force-generating mechanical transitions. In fact, it has been observed that ATP binding causes neck-linker movement towards the MT plus end^43, 44^. Although the free energy change associated with the ATP-induced neck-linker docking is small (about 3 kJ/mol)^44^ and only slightly larger than *k*_B_ *T* (~2.6 kJ/mol), it can create a forward bias for the mechanical step transition. A single-molecule experiment by Yildiz *et al*. demonstrated that ATP-free force-induced passive movements of kinesin can be achieved in the presence of ADP by much lower external forces than they can be in nucleotide-free conditions^45^. This observation suggests that the mechanical transition between states 1 (DE) and 4 (ED) would become important under forward and backward loads. Therefore, we considered the mechanical transitions between states 1 (DE) and 4 (ED) as well as between states 2 (DT) and 5 (TD) to construct kinesin’s chemomechanical network.

### ATP-free passive movement under loading

A chemomechanical network for ATP-free, force-assisted movements can easily be constructed using a simple three-state model, which, based on the arguments above, includes only the mechanical transitions between states 1 (DE) and 4 (ED) (Fig. 1b). The motor velocity is provided by ${v}_{14}=l\Delta {J}_{14}^{st}$, where *l* is the step size, i.e., 8 nm, and $\Delta {J}_{14}^{st}={J}_{14}^{st}-{J}_{41}^{st}$ is the steady-state excess flux between states 1 and 4. The steady-state local flux ${J}_{ij}^{st}$ is given by ${P}_{i}^{st}{\omega }_{ij}$ where ${P}_{i}^{st}$ is the time-independent steady-state probability and *ω*_*ij*_ is a transition rate from state *i* to state *j*.

In the absence of ATP, kinesin’s movement is a symmetric random walk irrespective of the ADP concentration as long as no external force is applied; thus, kinesin can produce no mechanical work. To make the network model satisfy the necessary condition of a nonequilibrium steady state, an extended steady-state balance condition (see Supplementary Information) was applied to the chemomechanical cycle, providing a relationship among the transition rates on the cycle 〈1471〉: ${\omega }_{14}^{0}{k}_{47}{\hat{k}}_{71}/{\omega }_{41}^{0}{k}_{17}{\hat{k}}_{74}=1$, where the definition of these parameters is provided at “Motor dynamics” in the Method section.

The chemical transitions between states 7 (EE) and 4 (ED) and between states 7 (EE) and 1 (DE) correspond to ADP binding to and release from the leading and trailing heads, respectively. These transitions are strongly affected by the intramolecular strain between the heads via the neck linkers^26^. Therefore, the rates for binding and release of ADP should be significantly different in the two heads; thus, asymmetries between ${\hat{k}}_{74}$ and ${\hat{k}}_{71}$ between *k*_47_ and *k*_17_ were introduced into the model.

### Unified eight-state model

Liepelt and Lipowsky^33^ proposed a six-state chemomechanical network model for kinesin, where states 7 (EE), 8 (TT), and 9 (DD) were omitted from the nine-state full representation (Fig. 1a) and only the mechanical transitions between states 2 (DT) and 5 (TD) were taken into account. They demonstrated that the model led to the motor properties that were in good agreement with the experimental data for the load dependence on the motor velocity, ratio of forward to backward steps, and randomness parameter at saturated and low ATP concentrations^33^. They also stated that the weakest binding state, state 9 (DD), and a subset of chemical transitions related to state 9 (DD) were required to describe the load and ATP-concentration dependences of the mean run length and the ADP-concentration dependence of the motor velocity^33^. Hyeon *et al*.^46^ and Lipelt and Lipowsky^38^ also considered the mechanical transitions between states 1 (DE) and 4 (ED) in their studies.

Our motivation in the study described herein was to develop a unified description of the chemomechanical coupling for ATP-dependent force-generating motility and ATP-free force-assisted passive movements. To achieve this purpose, we developed an eight-state chemomechanical network model (Fig. 1c). The necessity of the eight-state model is systematically discussed below. To include both ATP-dependent motility and ATP-independent force-assisted movement, we first combined the six-state model mentioned above and the three-state model shown in Fig. 1b into a seven-state model. This seven-state model, which is notably different from the seven-state model of Liepelt and Lipowsky^33^, corresponds to the eight-state model shown in Fig. 1c without state 8 (TT). Both of the mechanical transitions, specifically, the transitions between states 2 and 5 and between states 1 and 4, were taken into account. This seven-state model is useful for identifying asymmetries and similarities between the chemical transitions based on effects of the intramolecular strain on the chemical transitions. For example, we expect that the chemical transitions between states 5 (TD) and 6 (TE) should be similar to those between states 4 (ED) and 7 (EE), because these transitions correspond to ADP release from and binding to the leading head that is strongly pulled backward by the internal strain because the partner trailing head is strongly bound to the MT. In the same manner, the chemical transitions between states 2 (DT) and 3 (ET) should be similar to those between states 1 (DE) and 7 (EE). By assuming these similarities between the chemical transitions based on the intramolecular strain, the number of unknown parameters in the chemical transition rates can be reduced (see Supplementary Information). The similarity between the chemical transitions implies that the seven-state model must be extended to an eight-state model by including state 8 (TT) and the corresponding chemical transitions. In the seven-state model, the chemical transitions between states 3 (ET) and 7 (EE) and between states 6 (TE) and 7 (EE) play crucial roles for producing local fluxes through state 7 (EE) because the probability of state 7 (EE) becomes large at low ATP concentrations. According to the similarity relations based on intramolecular strain considerations, the chemical transitions between states 8 (TT) and 3 (ET) would be similar to those between states 6 (TE) and 7 (EE), and in the same way, the chemical transitions between states 8 (TT) and 6 (TE) would be similar to those between states 3 (ET) and 7 (EE). Therefore, we expect that these chemical transitions related to state 8 (TT) would also be important, especially at high ATP concentrations. It is expected that the omission of state 9 (DD) in the eight-state model might more strongly affect the detachment kinetics of kinesin from the MT than the motility of kinesin since state 9 (DD) is the weakest binding state to the MT. The details of the multiple unbinding processes considered in the present study to take into account effects of the detachment of kinesin from state 9 (DD) are explained in “Calculation details” of the Method section and in Supplementary Information. Below, we will show that the eight-state model that includes states 8 (TT) and 7 (EE) provides a unified description for available single-molecule observation data and also yields insights into the gating mechanisms.

## Results

### ATP-free passive movement under loading

**Figure 2:**
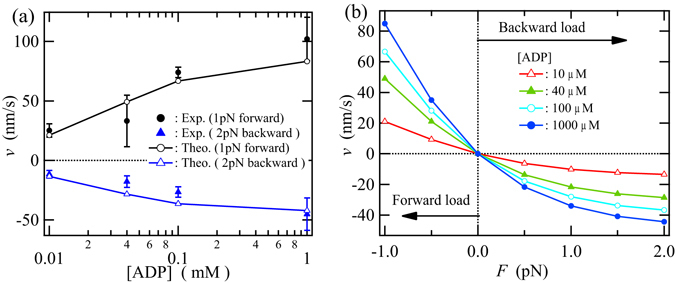
ATP-free force-assisted passive bidirectional movement of kinesin in the presence of ADP. (**a**) Comparison between the experimental data and the results provided by the three-state model (hereafter referred to as the theoretical results) for the ADP-concentration dependence of the motor velocity at 1 pN forward force and 2 pN backward force. The experimental data are taken from the literature^45^. (**b**) Theoretical results for the force dependence of the motor velocity at different ADP concentrations.

Figure 2a shows the ADP-concentration dependence of the motor velocity for ATP-free force-induced passive bidirectional movement of kinesin. The results provided by the three-state model agree well with the experimental data. Hereafter, the results obtained from the network model are basically referred to as the theoretical result. The determined parameters for the transition rates are listed in Table 1. The definition of the transition rate parameters and the detailed procedures on the determination of the parameters including those shown in Table 1 are provided at “Motor dynamics” and “Calculation details” in the Method section, respectively.

**Table 1:** Transition rates employed in the three-state network model shown in Fig. 1b.

Parameters	Meaning	Values
${\omega }_{14}^{0}$	Forward transition from state 1 to state 4	15.0 s^−1^
${\omega }_{41}^{0}$ ^*^	Backward transition from state 4 to state 1	13.5 s^−1^
*F*_14_	Force dependence on the 1–4 transition	0.747 pN
*θ*_14_	Load distribution between states 1 and 4	0.999
*k*_47_	ADP release from the leading head	550 s^−1^
${\hat{k}}_{74}$	ADP binding to the leading head	11.0 (μMs)^−1^
*k*_17_	ADP release from the trailing head	50.0 s^−1^
${\hat{k}}_{71}$	ADP binding to the trailing head	0.90 (μMs)^−1^

The definition of the parameters is provided at “Motor dynamics” in the Method section.

^*^Determined by the extended detailed balance condition (see Supplementary Information).

The force-assisted passive movement is obviously asymmetric as seen in Fig. 2b: the increase in the motor velocity toward the MT plus end with increasing forward force is larger than that toward the MT minus end with increasing backward force. The mechanical transition rates between states 1 (DE) and 4 (ED), ${\omega }_{14}^{0}$ and ${\omega }_{41}^{0}$, are almost the same, whereas the load distribution *θ*_14_ is very asymmetric, because these mechanical transitions should produce symmetric random walk as long as no external load is applied. On the other hand, the rate of ADP release from the leading head, i.e., the transition from state 4 (ED) to state 7 (EE), is about 10 times larger than that from the trailing head, i.e., the transition from state 1 (DE) to state 7 (EE). The absolute values of these ADP release rates determined by the network model during the nonequilibrium stepping processes seem to be different from those directly determined by biochemical experiment under thermodynamic equilibrium^47^, whereas the network model leads to the asymmetries between the chemical transition rates such that ADP release rate from the trailing head is smaller than that from the leading head, as observed by biochemical experiment^47^. The discrepancies between the absolute values of these rates can be explained based on the differences in the average structures and structure fluctuations of the motor domains between under thermodynamic equilibrium and during the nonequilibrium stepping processes. In fact, the large asymmetries between the chemical transition rates on the leading and trailing heads are basically attributable to the differences between the average structures of the motor domains in the leading and trailing heads due to the internal strain. In the same way as the ADP release rates, the rate of ADP binding to the leading head, i.e., transition from state 7 to state 4, is also about 10 times larger than the one to the trailing head, i.e., the transition from state 7 to state 1. The asymmetries between these chemical transition rates on the leading and trailing heads are caused by the intramolecular strain between two heads, while the asymmetric behavior between the ATP-free load-induced forward and backward passive movements is mainly attributable to the load-direction-dependent binding affinity of the head to the MT due to the asymmetric structure of kinesin’s head^26^, which is described by the load distribution parameter in the three-state model. It is noted that, according to the determination procedures of the transition rates, the validity of these ADP binding and release rate parameters employed in the three-state model is examined by applying to the transition rates between states 2 and 3 and between states 5 and 6 in the eight-state model based on the similarity relations among the chemical transitions and by comparing the theoretical results with the experimental motor velocities at various ATP and ADP concentrations, that are shown later in Fig. 4a and b.

### Stepping ratio and motor velocity for ATP-dependent force-generating motility

**Figure 3:**
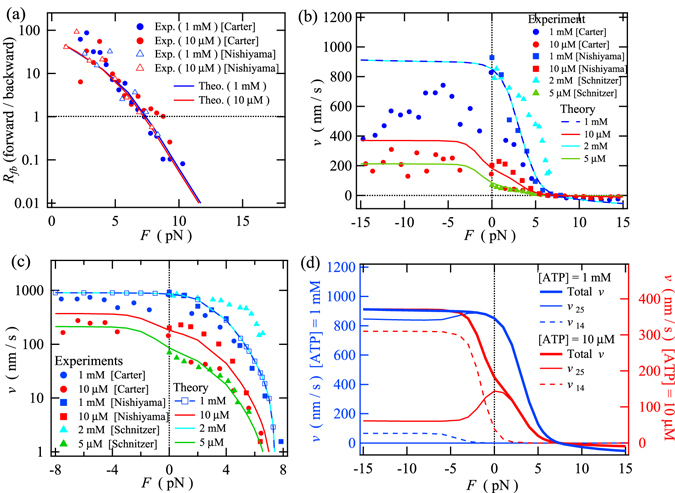
Force dependences of (**a**) the ratio of forward to backward steps, (**b**) and (**c**) the motor velocity [(**b**) and (**c**) show the same data, plotted on linear and logarithmic scales, respectively], and (**d**) contributions to the total velocity *v* from the steady-state excess local fluxes between states 2 and 5, ${v}_{25}=l{\rm{\Delta }}{J}_{25}^{st}$, and between states 1 and 4, ${v}_{14}=l{\rm{\Delta }}{J}_{14}^{st}$. The experimental data are from the literature^12, 48, 49^. In (**d**), the velocities at ATP concentrations of 1 mM and 10 μM are shown on the left and right axes, respectively.

The ratio of the number of forward steps to the number of backward steps depends on the external load but does not significantly depend on the ATP concentration (Fig. 3a). The stepping ratio shows the robustness of kinesin’s motility against increases in the external load and decreases in the ATP concentration. As discussed in the previous section, in the limit of infinite dilution of ATP, ADP-dependent passive movements have been observed by applying external forces much lower than the stall force and can be described by the three-state model where the mechanical transitions between states 4 (ED) and 1 (DE) are taken into account. On the other hand, it should be noted that kinesin generates forward forces as large as those under saturated ATP conditions, even if a very small amount of ATP is supplied (Fig. 3a). Therefore, even when the ATP concentration is much lower than the saturated ATP concentration, the probability of state 4 (ED) should always be much smaller than that of state 1 (DE) at any particular backward load, because the mechanical transition from state 4 (ED) to state 1 (DE) frequently occurs under backward loading if state 4 is occupied with high probability. The stepping ratio provides information necessary to determine unknown chemical transition rates in the eight-state network and to describe the response of the chemomechanical network to the changes in both the external force and the ATP concentration. If the force-assisted mechanical transitions between states 4 and 1 had not been taken into account in the eight-state model, we would not have noticed the importance of the highly asymmetric chemical transition pathways for the forward and backward stepping cycles that should avoid state 4 (ED) to inhibit frequent backward steps under backward loading.

The force dependence of the motor velocity is the other important characteristic of the motility of the molecular motor and can also be used to determine the unknown rates of the mechanical and chemical transitions. The results of several experimental studies showing the motor velocity as a function of the external force at various ATP concentrations^12, 48, 49^ are plotted in Fig. 3b and c and compared to the corresponding theoretical results calculated using the eight-state model. It is evident from Fig. 3c that the theoretical results agree well with the experimental data for the load dependence of the motor velocity under backward loading. It is also apparent that the eight-state model can describe the effect of the ATP concentration on the motor velocity during backward steps under superstall loading^12^; the backward velocity slightly increases as the ATP concentration increases under this type of loading (see Figs 3b and 2S in Supplementary Information). When an assisting force is present, the eight-state model predicts that the motor velocity will become saturated at any ATP concentration, even if the assisting force is increased (Fig. 3b). This theoretical prediction agrees well with recent experimental observations^50^. In addition, Fig. 3d demonstrates that the contribution to the total velocity from the individual excess fluxes depends on the ATP concentration. At low ATP concentrations, the main contribution to the total velocity when an assisting force is present arises from ${\rm{\Delta }}{J}_{14}^{st}$, i.e., *v*_14_, whereas almost the entire contribution under backward loading is from ${\rm{\Delta }}{J}_{25}^{st}$. i.e., *v*_25_ (see Fig. 3d). At saturating ATP concentrations, almost all of the contributions to the total velocity are attributable to ${\rm{\Delta }}{J}_{25}^{st}$, i.e., *v*_25_, independent of the direction of the external force. The force independence of the velocity when a strong assisting force is present (Fig. 3b) indicates that the rate-limiting transition should be attributable either to ATP hydrolysis on the trailing head or to ATP binding to the trailing head because the ADP-bound rear head, which is weakly bound to the MT, is necessary for the forward step transition to occur. If the ATP concentration is saturated, the rate-limiting transition must be the ATP hydrolysis on the trailing head because the rate of ATP binding is sufficiently large. On the other hand, if the ATP concentration is low, the rate-limiting transition is attributable to ATP binding to the trailing head, which is followed by the ATP hydrolysis reaction. These observations will be discussed in detail below on the basis of the excess fluxes on the network.

### ADP effects on the motor velocity

**Figure 4:**
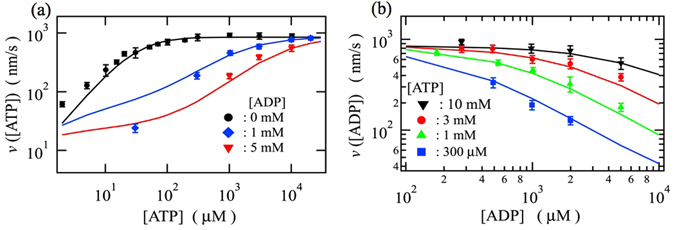
(**a**) ATP-concentration dependences of the motor velocity at several ADP concentrations. (**b**) ADP-concentration dependences of the motor velocity at several ATP concentrations. The lines indicate the theoretical results and the symbols indicate the experimental data obtained from the literature^23^.

We can expect that the chemical transitions related to ADP binding and release would play a crucial role in ADP effects on the motor velocity. Figure 4 shows (a) ATP-concentration dependences of the motor velocity at several ADP concentrations and (b) ADP-concentration dependences of the motor velocity at several ATP concentrations. The eight-state model, where the rate constants employed in the three-state model (Table 1) are also applied to the transition rates between states 2 and 3 and between states 5 and 6 based on the similarity relations, can describe the ADP effects on the motor velocity at varying ATP concentrations. These results show the validity of the transition rates between states 1 and 7 and between 4 and 7 employed in the three-state model to describe the ATP-free load-assisted passive movements shown in Fig. 2a. In Fig. 4a, we can observe an ADP effect that suppresses the increase in the motor velocity with increasing ATP concentration. Figure 4b shows that the motor velocity is decreased by the increase in the ADP concentration. These observations indicate that ATP binding to the leading and/or trailing head becomes an additional rate-limiting process in the forward stepping cycle as ADP concentration increases since competitions between ATP binding and ADP binding to the leading and/or trailing head occur. The results of the excess local fluxes, that are shown later in Fig. 6a and b, imply that the ATP binding transitions to the leading and trailing heads become an rate-limiting at high and low ATP concentrations, respectively, mainly due to the ADP binding transitions to the leading head (the 6-to-5 and 7-to-4 transitions, respectively) under high ADP concentration because the ADP binding rates to the leading head are about ten times larger than the ones to the trailing head [see Table 1].

### Unbinding rate and mean run length

**Figure 5:**
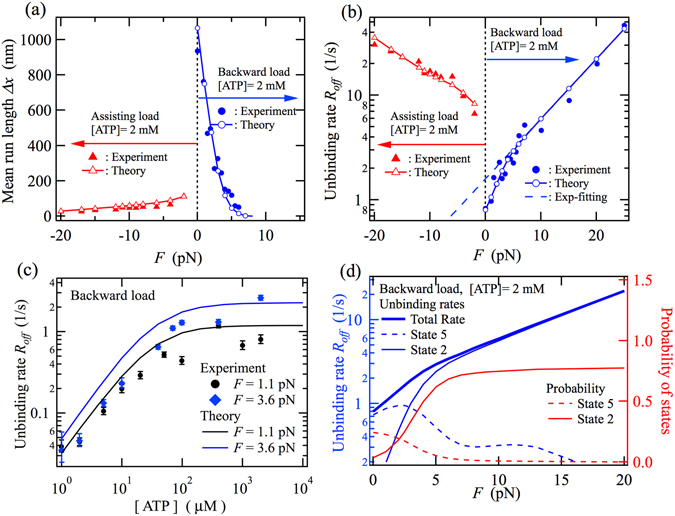
Force dependences of (**a**) mean run length Δ*x* and (**b**) unbinding rate *R*_*off*_ at a saturated ATP concentration of 2 mM. (**c**) ATP-concentration dependence of *R*_*off*_ at backward loads of 1.1 pN and 3.6 pN. (**d**) (Left axis) Backward load dependence of partial unbinding rates from states 2 and 5 at 2 mM ATP; (right axis) the backward load dependence on the probabilities of states 2 and 5 at an ATP concentration of 2 mM. The experimental data shown in Fig. 5a and b are from the literature^50^. The experimental unbinding rates shown in Fig. 5c were calculated using ${R}_{off}=v/{\rm{\Delta }}x$, with the experimental data^48^. In Fig. 5b, the broken line shows an exponential fit to the theoretical results under backward loads greater than 10 pN.

Intracellular transport of cargo by molecular motors can be characterized by the mean run length Δ*x* and corresponding unbinding rate *R*_*off*_. To calculate these detachment properties of kinesin from the MT, the eight-state model shown in Fig. 1c is extended. The calculation details of the detachment properties are explained in “Calculation details” of the Method section and in Supplementary Information. As shown in Fig. 5a, the mean run length exhibits an asymmetric dependence on the direction of the applied load and seems to decrease exponentially as the absolute value of the external load increases^50, 51^. In Fig. 5b, the load dependence of the unbinding rate of kinesin from MT is shown on a semi-logarithmic scale. The asymmetric load dependence of the unbinding rate is consistent with the direction-dependent unbinding force distribution of kinesin’s single head from the MT^26^. The data obviously deviate from the exponential fit, which is shown as the blue broken line in Fig. 5b, for backward loads less than about 7 pN. The non-exponential behavior implies that multiple pathways would contribute to unbinding via different states and that the related states would occur with load-dependent probabilities. In Fig. 5c, the ATP-concentration dependence of the unbinding rate is shown for constant backward loads of 1.1 pN and 3.6 pN. The increase in the unbinding rate with increasing ATP concentration shows that the states whose probabilities are large at high concentrations of ATP have larger rates of unbinding from the MT. In addition to multiple unbinding processes via different states, dependence on the ATP concentration of the probabilities of the related states might be necessary to describe the ATP-dependent unbinding rate.

To clarify the non-exponential behavior of the unbinding rate under backward loading, a comparison of the partial unbinding rates from states 2 and 5 with their probabilities is shown in Fig. 5d. The contribution from state 5 to the total unbinding rate is dominant when there is no backward load. This result can be qualitatively interpreted as follows: state 5 is fragile against backward loading since the leading head is weakly bound to the MT. As the backward load increases, the contribution from state 5 starts to decrease around 2.5 pN since the probability of state 5 decreases, while the contribution from state 2 to the total unbinding rate increases and becomes dominant at loads larger than about 7 pN, where the probability of state 2 becomes larger than 0.7.

### Chemical transition pathways at different ATP concentrations and external forces

**Figure 6:**
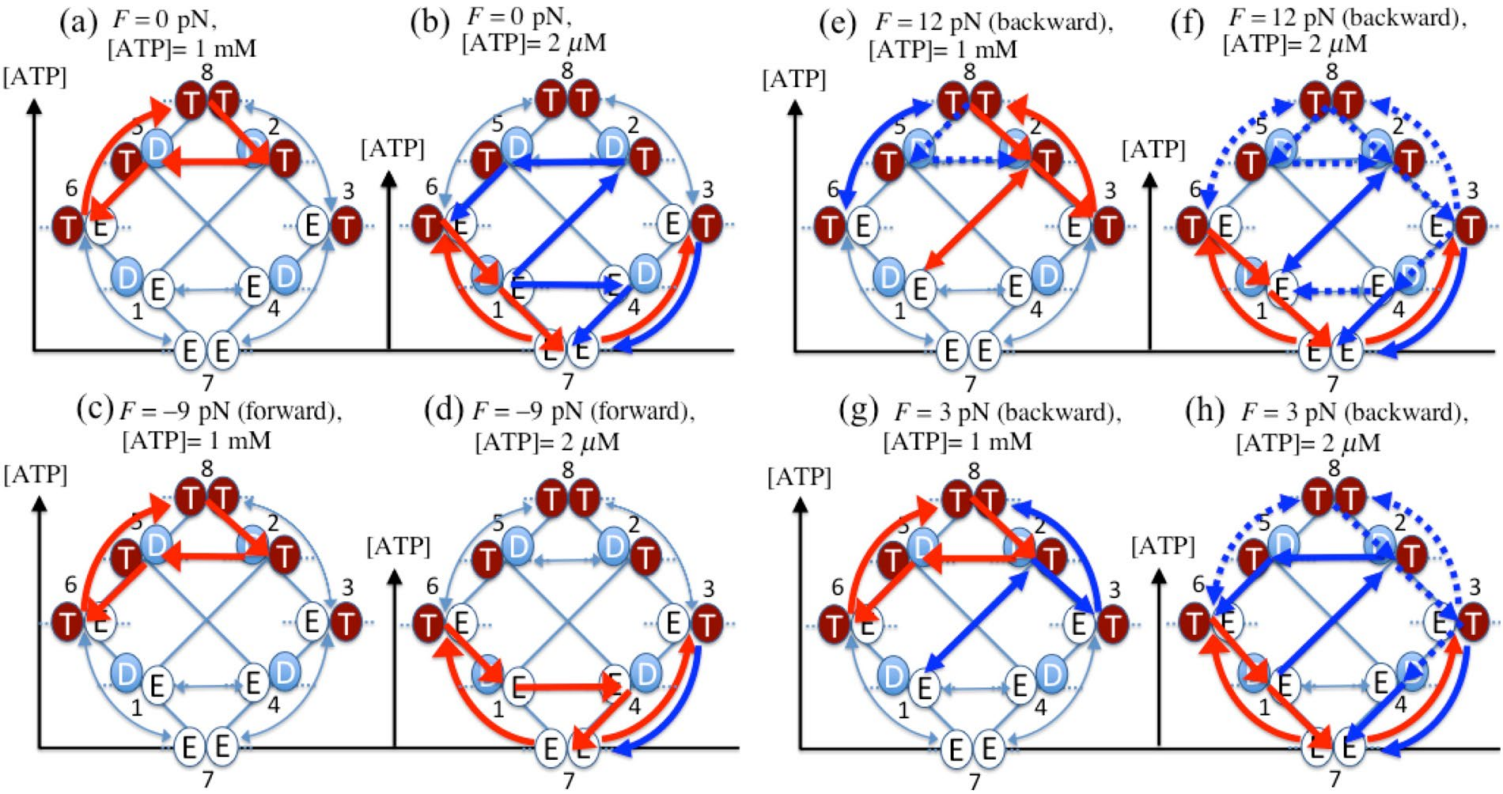
Diagram of main local fluxes corresponding to high (1 mM) and low (2 μM) ATP concentrations (**a**,**b**) without a load and (**c**,**d**) with an assisting (forward) force of 9 pN and backward loads of (**e**), (**f**) 12 pN and (**g**), (**h**) 3 pN. The red arrows indicate normalized local fluxes larger than 0.10, while the blue arrows indicate the other normalized local fluxes larger than 0.02, where the normalized local flux is the local flux divided by the sum of all of the local fluxes. The dashed blue arrows indicate normalized local fluxes less than 0.02 but are directly related to the mechanical transitions. Bidirectional arrows signify that the absolute values of both of the local fluxes are almost the same so that the excess flux is about zero.

Most of the questions about the chemomechanical coupling of kinesin presented in the introduction can be answered by investigating the local fluxes at several ATP concentrations and external forces. Figure 6a–d show the normalized local fluxes without a load and for an assisting force of 9 pN, where the normalized local flux is defined as the value of the local flux divided by the sum of all of the local fluxes. Comparison of these figures clearly shows that the forward stepping cycle strongly depends on the ATP concentration. When the ATP level is saturated (Fig. 6a and c), the forward stepping cycle, which is equivalent to the main local flux, is located in the upper half side of the network, independent of whether or not an assisting force is applied. On the other hand, when the ATP level is low, the main local flux, shown by the red arrows in Fig. 6b and d, is located in the bottom half of the network, while the forward stepping cycle depends on the external force. The excess fluxes related to the forward stepping cycle are located only in the bottom half of the network in the case in which an assisting force was applied (Fig. 6d) but are spread over both sides of the network in the case in which no assisting force was applied (Fig. 6b). The ATP-dependent forward stepping cycle would never be observed if states 7 (EE) and 8 (TT) were omitted as done by Liepelt and Lipowsky^33^. When the ATP level is saturated, the probability of state 8 (TT) is about 0.7 both without an assisting force and with a 9 pN assisting force, and the force-generating mechanical transition from state 2 (DT) to state 5 (TD) occurs quickly upon ATP hydrolysis on the trailing head. Therefore, the transition from state 8 (TT) to state 2 (DT), i.e., ATP hydrolysis on the trailing head, is the rate-limiting transition. On the other hand, when the ATP level is low, the probability of state 7 (EE) is 0.74 without an assisting force and 0.86 with a 9 pN assisting force; thus, the transition from state 7 (EE) to state 6 (TE), i.e., ATP binding to the trailing head, is the rate-limiting transition. When the ATP level is low, if no assisting force is applied (Fig. 6b), kinesin uses the force-generating mechanical transition from state 2 to state 5 and the non-power stroke transition from state 1 to state 4. Indeed, the local flux from state 1 to state 4 $({\rm{\Delta }}{J}_{14}^{st}=2.16\,{{\rm{s}}}^{-1})$ is larger than that from state 1 to state 2 $({\rm{\Delta }}{J}_{12}^{st}=1.15\,{{\rm{s}}}^{-1})$. Although the transition rate from state 1 to state 4 is almost the same as that from state 4 to state 1, as shown in Table 1, the non-power stroke mechanical transition from state 1 (DE) to state 4 (ED) is possible because the probability of state 1 is a few hundred times higher than that of state 4. In contrast to the power-stroke transition from state 2 to state 5, the rate of the non-power stroke transition from state 4 to state 1 is 200 times smaller; thus, kinesin might appear to be in a one-head-bound state during the transition from state 4 to state 1 in single-molecule observations.

Figure 6e–h show the local fluxes corresponding to applied backward loads of 3 pN and 12 pN. First, we compare the local fluxes obtained by applying a backward load of 12 pN (Fig. 6e) to those obtained without applying an external load (Fig. 6a). When the ATP level is saturated, kinesin’s network changes the locus of the main local flux from upper left to upper right in the diagram as the backward load is increased to the superstall load. On the other hand, when the ATP level is low, the main local flux when a superstall load is applied (indicated as the red arrows in Fig. 6f) remains in the bottom left region. These observations imply that the kinesin network avoids states 4 (ED) and 5 (TD) as much as possible during the backward stepping cycle because the backward steps spontaneously occur from those fragile states where the leading head is weakly bound to the MT. For the cases in which 3 pN backward loads were applied (Fig. 6g and h), the main local fluxes for both high and low ATP concentrations are almost equal to the averages of those without a backward load and with the application of a 12 pN backward load.

## Discussion

### Gating mechanism

Models of chemical transition pathways for molecular motors are often based on the single cycles through which the motor proceeds in a straightforward cyclic manner. By contrast, in the present study, we avoided such an intuitive reduction of the state space related to chemical and mechanical transitions. Instead, to study the mechanochemistry of the kinesin motor, we systematically introduced similarity relations among transition rates connecting similar states, as well as asymmetries reflecting differences between the specific processes on the two heads. These relationships between the rates are based on the intramolecular strain between the two heads. As a result, we attained an eight-state model that can quantitatively describe the available experimental data, as shown in Figs 2, 3, 4 and 5. Furthermore, when the ATP concentration was saturated, the eight-state model provided the high ratio of the number of ATP hydrolyses to the number of advanced steps, indicating a tight coupling between ATP hydrolysis and the mechanical step^14–16^ (see Fig. S3), even though the eight-state model contained many chemical transition pathways that might have resulted in futile cycles. The gating mechanism that was necessary to yield the extraordinary motor properties of kinesin was attained only through the structure of the chemomechanical network and the asymmetric chemical transition rates caused by the intramolecular strain between the two heads.

Based on the analysis of the local fluxes in the nonequilibrium steady state, we found that the chemical transition pathways for forward stepping strongly depended on both the ATP concentration and the external load. When the ATP level was saturated, the eight-state model showed a gated rear head mechanism for the forward stepping cycle, regardless of whether a forward or backward force (less than the stall force) was applied. In these cycles, ATP hydrolysis on the trailing head was a rate-limiting transition and was thus followed by the force-generating mechanical transition from state 2 (DT) to state 5 (TD) (Fig. 6a,c and g). Kinesin, therefore, spent most of its time bound to the MT with both heads. The gated rear head mechanism that occurred when the ATP level was saturated basically agreed with one of the models proposed by Mori *et al*. based on smFRET experiments^18^. They noted two models for the forward stepping cycle when the ATP level was saturated: one that includes ATP hydrolysis from state 8 (TT) to state 2 (DT), followed by the mechanical transition from state 2 (DT) to state 5 (TD), and another that includes ATP hydrolysis from state 6 (TE) to state 1 (DE) and the mechanical transition from state 1 (DE) to state 4 (ED). Notably, the 1-to-4 transition is assumed to be a power stroke transition in the case of Mori *et al*.’s latter model because they observed only a two-head-bound intermediate between steps when the ATP level was saturated. The eight-state model strongly suggests the former proposal and is not consistent with the latter proposal. In fact, if the 1-to-4 transition were a power stroke transition, the one-head-bound state would never have been observed because the power stroke transition spontaneously transforms state 1 (DE) into state 4 (ED) as soon as ATP hydrolysis on the trailing head (i.e., the 6-to-1 transition) occurs. As explained below, this assumption in the latter proposal would not be consistent with the model Mori *et al*. proposed for low ATP concentrations.

When the ATP level was low, in which cases ATP binding was the rate-limiting transition, the eight-state model showed a load-dependent gating mechanism for forward stepping cycles and the common main excess flux corresponding to a futile cycle of ATP hydrolysis on the trailing head. When no load was present, the two kinds of mechanical transitions were gated by ATP hydrolysis on the trailing head and by ATP binding to the leading head (Fig. 6b). On the other hand, under backward loading, the eight-state model showed that kinesin used only the gated front head mechanism, in which the force-generating mechanical transition from state 2 (TD) to state 5 (TD) was regulated by ATP binding to the leading head (Fig. 6h). The transition pathway gated by ATP binding to the leading head was consistent with the model of Mori *et al*. when the ATP level was low^18^. They suggested that kinesin waits for ATP binding to the leading head in a one-head-bound state and makes a power stroke transition to a two-head-bound state so that it walks on the MT^18^. On the contrary, the eight-state model shows a futile cycle of ATP hydrolysis on the trailing head as the main local flux (Fig. 6b) and also shows that the power stroke transition from state 2 (DT) to state 5 (TD) and the non-power stroke transition from state 1 (DE) to state 4 (ED) contribute to the forward stepping cycle. Here, if we consider the (ADP-bound) trailing head as being detached from the MT rather than weakly bound to the MT, the futile cycle of ATP hydrolysis on the trailing head would result in repeated unbinding and rebinding of the trailing head to the MT. Those behaviors might be regarded as the one-head-bound state of kinesin, which would be qualitatively consistent with Mori *et al*.’s experimental observations^18^. The cycle of unbinding and rebinding of the trailing head to the MT is necessary for the backward steps observed under superstall loads when the ATP level was low^12, 49^. This is because kinesin cannot directly make a backward step from the one-head-bound state.

### Forward stepping cycle assisted by external force

The force-assisted forward stepping cycle was also strongly dependent on the ATP concentration. When the ATP level was saturated, the forward stepping cycles assisted by external forces were basically the same as those without loads and with backward loads smaller than the stall force, while the behavior was slightly different when the ATP level was low. When the ATP level was saturated, the force-assisted forward stepping cycle went through the mechanical transition from state 2 (DT) to state 5 (TD), i.e., the power-stroke transition (Fig. 6c), while when the ATP level was low, it occurred via the non-power stroke mechanical transition from state 1 (DE) to state 4 (ED) (Fig. 6d). These force-assisted forward stepping cycles were regulated by a gated rear head mechanism due to ATP hydrolysis on the trailing head. The saturation of the motor velocity when a sufficiently large assisting force was applied was attributable to rate-limiting ATP hydrolysis when the ATP level was saturated and rate-limiting ATP binding to the trailing head when the ATP level was low, in addition to ATP hydrolysis.

### Chemical transition pathways for backward stepping cycle

The backward stepping cycle was completely different from the reverse of the forward stepping cycle and did not generate ATP, but rather consumed ATP. The main excess fluxes, as shown with the red arrows in Fig. 6e and f, depended strongly on the ATP concentration; however, the backward stepping cycles forced by external loads were identical when the ATP concentration was low and when it was high, i.e., |52385〉, suggesting that kinesin used one dominant mechanism for backward steps, causing the strong force dependence of the ratio of forward to backward steps. Kinesin’s network responded to superstall forces in such a way that the main excess flux always went through the states in which either both heads, or at least the leading head, were strongly bound to the MT. Otherwise, backward steps would be favored by a weakly bound leading head. Therefore, the ATP hydrolysis energy was consumed during the load-induced backward stepping cycles to restrain the diffusion of excess fluxes over the network and to direct excess fluxes to circumvent the states that were fragile when backward loads were applied.

## Summary

In this report, we presented a systematic model of molecular motor kinesin based on the chemomechanical network theory^33^. In the nine-state network representation (Fig. 1a), there were six possible mechanical transitions: the transitions between states 8 (TT) and 8 (TT), between state 9 (DD) and 9 (DD), between states 7 (EE) and 7 (EE), between states 2 (DT) and 5 (TD), between states 3 (ET) and 6 (TE), and between states 1 (DE) and 4 (ED) (Fig. 1a and c). We can exclude the transition between states 9 (DD) and 9 (DD) since both of the heads were weakly bound to the MT; thus, state 9 (DD) was easily unbound from the MT. Likewise, three transitions can be removed because both heads were strongly bound to the MT; thus, the corresponding mechanical steps hardly occurred. The two remaining transitions, between states 2 (DT) and 5 (TD) and between states 1 (DE) and 4 (ED), satisfied the necessary conditions for producing the mechanical steps; one head should be strongly bound to the MT, while the other head should be weakly bound to the MT. Therefore, only transitions between states 2 (DT) and 5 (TD) and between states 1 (DE) and 4 (ED) were taken into account as the mechanical step transitions in the eight-state model (Fig. 1c), in which state 9 (DD) was removed from the nine-state full representation. ATP-dependent force-generating processive motility^12, 48^ and ATP-free force-assisted passive movements^45^ were previously observed in single-molecule experiments. We expected that the former should be mainly coupled to the mechanical transitions between states 2 (DT) and 5 (TD) because ATP was needed by kinesin, while the later should be mainly connected to the transitions between states 1 (DE) and 4 (ED). As a result, the eight-state model showed that the main forward stepping cycle proceeded via state 2 (DT) to generate the power stroke mechanical step from state 2 (DT) to state 5 (TD) and also avoided state 4 (DE) to inhibit backward steps [state 4 (ED) to state 1 (DE)]. These results suggest that the ATP hydrolysis energy was used such that (i) kinesin’s network concentrated the chemical transition flux to the chemical state that can produce the power stroke transition from state 2 (DT) to state 5 (TD) when the ATP concentration was saturated and, (ii) when the ATP level was low, it avoided the states that were fragile when backward loads were applied, i.e., state 4, because of the weak binding of the leading head to the MT to prevent frequent backward stepping from state 4 (ED) to state 1 (DE).

## Methods

### Motor dynamics

The motor dynamics can be described by a continuous-time Markov process. Thus, the probability *P*_*i*_(*t*) of finding the motor in state *i* at time *t* is governed by the following master equation:

1 $$\frac{d}{dt}{P}_{i}(t)=-\sum _{j}\Delta {J}_{ij}(t),$$

where

2 $$\Delta {J}_{ij}(t)={P}_{i}(t){\omega }_{ij}-{P}_{j}(t){\omega }_{ji}$$

and *ω*_*ij*_ is a transition rate from state *i* to state *j*, i.e., the number of transitions from *i* to *j* per unit time, and *ΔJ*_*ij*_(*t*) is the local excess flux due to the transition from state *i* to state *j*. In general, *ω*_*ij*_ depends on both the external force parallel to the MT, *F*, and the molar concentrations [*X*], where *X* denotes the molecular species ATP, ADP, or Pi (inorganic phosphate). Thus, the transition rates can be calculated using

3 $${\omega }_{ij}={\omega }_{ij}^{0}{\Phi }_{ij}(F),$$

where ${\omega }_{ij}^{0}$ is the zero-force transition rate and *Φ*_*ij*_(*F*) is the force-dependent factor with *Φ*_*ij*_(*F* = 0) = 0. Moreover, ${\omega }_{ij}^{0}$ for the binding of an *X*-molecule depends on the molar concentration [*X*]:

4 $${{\omega }}_{ij}^{0}=\{\begin{array}{cc}{\hat{k}}_{ij}[X] & {\rm{for}}\,{\mbox{{\it X}-{{binding}}}}\\ {k}_{ij} & {\rm{for}}\,{\mbox{{\it X}-release}},\end{array}$$

where ${\hat{k}}_{ij}$ has dimensions of 1/μMs, while *k*_*ij*_ has dimensions of 1/s. In the present study, we assumed that only the mechanical transition rates depended on the external force and that *Φ*_*ij*_(*F*) for the forward step transitions when backward forces were applied had the following form (note that a backward force *F* is taken to be positive):

5 $${\Phi }_{ij}(F)=\exp [-F{\theta }_{ij}/{F}_{ij}]$$

and

6 $${\Phi }_{ji}(F)=\exp [F(1-{\theta }_{ij})/{F}_{ij}],$$

where *F*_*ij*_ is a force scale with respect to the force dependence of the mechanical transition and *θ*_*ij*_ is a load distribution factor with a value of 0 < *θ*_*ij*_ < 1.

### Calculation details

In the eight-state model, the motor velocity is provided by *v* = *v*_25_ + *v*_14_ with ${v}_{25}=l\Delta {J}_{25}^{st}$ and ${v}_{14}=l\Delta {J}_{14}^{st}$. There are 32 transitions among the eight states. In the chemomechanical network theory^33^, the steady-state balance condition^34^ is basically applied. Alternatively, in this study, an extension of the steady-state balance condition was applied to satisfy the necessary condition for a nonequilibrium steady state (see Supplementary Information). By using the extended steady-state balance condition and the similarities between the chemical transitions, the number of independent unknown transition rates could be reduced from 32 to 14 (see Supplementary Information). The determination of unknown parameters for the independent transitions was manually performed as follows. First, we applied the three-state model shown in Fig. 1b to the experimental motor velocities for the ATP-free force-assisted passive bidirectional movements at different ADP concentrations^45^ (Fig. 2a) and then determined the chemical and mechanical transition rates. Next, we applied the eight-state model (Fig. 1c with those parameters determined using the three-state model) to the experimental data for the external load dependences of the step ratio (Fig. 3a) and of the motor velocity (Fig. 3b and c) in the limit of infinite dilution of both ADP and Pi. We then checked all of the determined parameters by applying ADP concentration dependence to the motor velocity without an external load (see Fig. 4). If the agreement with all of the experimental data of the motor properties considered in this study was insufficient, these procedures including the ATP-free force-assisted passive bidirectional movements shown in Fig. 2a were repeated to refine the parameters until sufficient agreement with all the experimental data was achieved. After we determined all of the parameters, we applied an extended eight-state model in which unbinding transitions of kinesin from the MT via states 2 (DT), 5 (TD), 1 (DE), and 4 (ED) were taken into account (see Supplementary Information) for the experimental mean run length data (Fig. 5a). Finally, we recalculated all of the motor properties taking into account the unbinding transitions and refined all of the parameters by fitting the extended eight-state model to all of the experimental data again, indicating a kind of global fitting. It was found that the effect of the unbinding transitions on the motor velocity was small.

## Electronic supplementary material

Supplementary Information
