# Supplementary material for: Design principles governing chemomechanical coupling of kinesin
Source: Sci Rep. 2017 Apr 25;7:1163. doi: 10.1038/s41598-017-01328-9 (PMC5430765; doi:10.1038/s41598-017-01328-9)
Supplement: Supplementary file 1 — Supplementary Information [file 41598_2017_1328_MOESM1_ESM.pdf]

Supplementary information

Design principles governing chemomechanical coupling of kinesin

Tomonari Sumi[1,2]

[1]Research Institute for Interdisciplinary Science, Okayama University, 3-1-1 Tsushima-Naka, Kita-ku, Okayama 700-8530, Japan
[2]Department of Chemistry, Faculty of Science, Okayama University, 3-1-1 Tsushima-Naka, Kita-ku, Okayama 700-8530, Japan

An extension of the steady state balance condition

The steady state balance condition has been presented by Liepelt and Lipowsky [1] as a generalization of the detailed balance condition in equilibrium which would provide the necessary condition in nonequilibrium steady state for nanomachine. In their formulation, the first law of thermodynamics is applied to individual cycles and the second law with the equality for reversible process is used to relate the statistical entropy produced by the individual cycle to the heat released from the system. However, in my understanding, we have to apply the second law of thermodynamics based on both the equality and inequality to characterize irreversible cycles of nanomachine in nonequilibrium steady state. Let's start with the conservation of energy during the completion of individual cycle according to the formulation presented by Liepelt and Lipowsky. [1] A change in the internal energy $\Delta U\left(C_\nu^+\right)$ along any directed cycles C_ν^+ satisfies

$$\Delta U\left(C_\nu^+\right)=E_{chem}\left(C_\nu^+\right)-W_{mech}\left(C_\nu^+\right)-Q\left(C_\nu^+\right), \tag{S1}$$

where $E_{chem}\left(C_\nu^+\right)$ is the energy input given by chemical reactions, $W_{mech}\left(C_\nu^+\right)$ mechanical work performed by mechanical transitions against an external load, and $Q\left(C_\nu^+\right)$ heat released from the system, during the completion of the directed cycle C_ν^+.

In this study, we assume that the motor dynamics is described by a continuous-time Markov process with transition rates ω_{ij} from state *i* to state *j*. The statistical entropies $\Delta S\left(C_\nu^+\right)$ and $\Delta S\left(C_\nu^-\right)$, which are produced in the steady state during the completion of directed cycles C_ν^+

and C_v^-, are provided by [1,2]

$$\Delta S\left(C_v^+\right) = k_B \sum_{|ij\rangle}^{v,+} \ln\left(\omega_{ij}/\omega_{ji}\right) \equiv -\Delta S\left(C_v^-\right), \tag{S2}$$

where the summation is performed over all edges $|ij\rangle$ along the directed cycle C_v^+. Now we apply the second law of thermodynamics to relate the statistical entropy $\Delta S\left(C_v^+\right)$ to the heat released from the system:

$$\Delta S\left(C_v^+\right) \geq Q\left(C_v^+\right)/T, \tag{S3}$$

where the equality holds if the directed cycle C_v^+ is reversible. Using Eqs. (S1)-(S3), we obtain the following equation:

$$\left\{E_{chem}\left(C_v^+\right) - W_{mech}\left(C_v^+\right)\right\}/k_B T - \sum_{|ij\rangle}^{v,+} \ln\left(\omega_{ij}/\omega_{ji}\right) \leq 0, \tag{S4}$$

which can be regarded as an extended steady state balance condition where only the inequality is different from that originally presented by Liepelt and Lipowsky. [1] The irreversibility of the directed cycles resulting in the inequality of Eq. (S4) should be related to the non-zero flux J_v^+ for the directed cycle C_v^+ and/or the non-zero J_v^- for its reversed cycle C_v^-. In the case of molecular motor kinesin, the irreversibility of these directed cycles would actually be reflected on the difference between the forward main cycle and the backward main cycle under superstall load.

Here the chemical energy input per directed cycle can be expressed as

$$E_{chem}\left(C_v^d\right) = \left[n_{\mathrm{h}}\left(C_v^d\right) - n_{\mathrm{s}}\left(C_v^d\right)\right]\Delta\mu, \tag{S5}$$

$$\Delta\mu = k_B T \ln\left\{\left(K_{eq}[\mathrm{ATP}]\right)/\left([\mathrm{ADP}][\mathrm{P}]\right)\right\}, \tag{S6}$$

where $n_{\mathrm{h}}\left(C_v^d\right)$ and $n_{\mathrm{s}}\left(C_v^d\right)$ are respectively the numbers of hydrolysis and synthesis that are contained in one directed cycle of C_v^d, and $\Delta\mu$ is the excess free energy change that depends on the concentrations of [ATP], [ADP], and [P], and is given by one ATP hydrolysis, resulting in binding one ATP to the motor and releasing one ADP and one P from the motor, where the standard state for the change in $\Delta\mu$ is the chemical equilibrium with the equilibrium constant K_{eq}.[3,4] In the presence of an external load *F*, the mechanical work performed by the molecular motor during the completion of the directed cycle C_v^d is given by

$$W_{mech}\left(C_v^d\right) = \left[m_{\mathrm{f}}\left(C_v^d\right) - m_{\mathrm{b}}\left(C_v^d\right)\right] lF, \tag{S7}$$

where l is the size of the mechanical step and $m_{\mathrm{f}}\left(C_{v}^{d}\right)$ and $m_{\mathrm{b}}\left(C_{v}^{d}\right)$ are respectively the numbers of the mechanical forward and backward steps that are contained in one directed cycle of C_{v}^{d}. Substituting Eqs. (S5) and (S7) into Eq. (S4), we obtain

$$\left\{\left[n_{\mathrm{h}}\left(C_{v}^{+}\right)-n_{\mathrm{s}}\left(C_{v}^{+}\right)\right]\Delta\mu-\left[n_{\mathrm{f}}\left(C_{v}^{+}\right)-n_{\mathrm{b}}\left(C_{v}^{+}\right)\right]Fl\right\}/k_{B}T-\sum_{|ij\rangle}^{v,+}\ln\left(\omega_{ij}/\omega_{ji}\right)\leq 0\,. \tag{S8}$$

Now we assume that the transition rates are given by Eqs. (3)-(6) in the text. To extract information from Eq. (S8), we consider the case of F=0 where the force dependent factors $\Phi_{ij}\left(F\right)$ vanish and thus the following equation is obtained:

$$\left[n_{\mathrm{f}}\left(C_{v}^{+}\right)-n_{\mathrm{b}}\left(C_{v}^{+}\right)\right]\Delta\mu/k_{\mathrm{B}}T-\sum_{|ij\rangle}^{v,+}\ln\left(\omega_{ij}^{0}/\omega_{ji}^{0}\right)\leq 0\,. \tag{S9}$$

If the excess fluxes $\Delta J_{v}^{+}s$ for all the directed cycles are equal to zero, the system is in thermodynamic equilibrium, thus all the directed cycles are reversible and then the equality in Eq. (S9) should hold. As a result, we obtain

$$\sum_{|ij\rangle}^{v,+}\ln\left(\omega_{ij}^{0}/\omega_{ji}^{0}\right)=\left[n_{\mathrm{f}}\left(C_{v}^{+}\right)-n_{\mathrm{b}}\left(C_{v}^{+}\right)\right]\Delta\mu/k_{\mathrm{B}}T=0\,, \tag{S10}$$

which corresponds to the detailed balance conditions in equilibrium presented by Liepelt and Lipowsky.[1] It has been pointed out by them that the detailed balance conditions in equilibrium are fully satisfied, if Eq. (S10) is applied to all the fundamental cycles.

Next, we substitute Eqs. (3) and (4) in the text and Eq. (S9) into Eq. (S8), and then we obtain the following condition under the external load:

$$\sum_{|ij\rangle}^{v,+}\ln\left(\Phi_{ij}(F)/\Phi_{ji}(F)\right)\geq-\left[m_{\mathrm{f}}\left(C_{v}^{+}\right)-m_{\mathrm{b}}\left(C_{v}^{+}\right)\right]Fl/k_{\mathrm{B}}T\,, \tag{S11}$$

where the equality holds in reversible process. In the case that all the directed cycles in the system include the mechanical transitions, where a single cycle system is a typical case, at the stall load condition, i.e., $\Delta J_{v}^{+}=0$ for all the directed cycles, the system should be in thermal and mechanical equilibrium. In this case, the directed cycles are reversible and thus the equality in Eq. (S11) should hold. As a result, we obtain

$$\sum_{|ij\rangle}^{v,+}\ln\left(\Phi_{ij}(F)/\Phi_{ji}(F)\right)=-\left[m_{\mathrm{f}}\left(C_{v}^{+}\right)-m_{\mathrm{b}}\left(C_{v}^{+}\right)\right]Fl/k_{\mathrm{B}}T\,, \tag{S12}$$

which is equivalent to a part of the steady state balance condition presented by Liepelt and Lipowsky,[1] and gives a relation $1/F_{ij}=l/k_{\mathrm{B}}T$ by substituting Eqs. (5) and (6) in the text into Eq. (S12). On the other hand, in the case that the system has the directed cycles without the mechanical transitions, i.e., futile chemical cycles, even at the stall load conditions, i.e., $\Delta J_{v}^{+}=0$ only for all the directed cycles including the mechanical transitions, the futile chemical cycles would be driven by the energy input.

In this case, the system is in nonequilibrium and the directed cycles would be irreversible, and thus the inequality should hold in Eq. (S11). As a result, we obtain the following condition:

$$\sum_{|ij\rangle}^{\nu,+} \ln\left(\Phi_{ij}(F)/\Phi_{ji}(F)\right) > -\left[m_{\rm f}\left(C_\nu^+\right) - m_{\rm b}\left(C_\nu^+\right)\right] Fl \Big/ k_{\rm B}T \,. \qquad \text{(S13)}$$

In our model, Eq. (S13) is satisfied by the condition $1/F_{ij} < l/k_{\rm B}T$ that is provided by substituting Eqs. (5) and (6) in the text into Eq. (S13). To satisfy the extended steady state balance condition provided by Eq. (S4), we impose the detailed balance conditions in equilibrium, i.e., Eq. (S10), to zero-force transition rates ω_{ij}^0 by Eq. (4) in the text and also apply the condition $1/F_{ij} < l/k_{\rm B}T$ that is provided by Eq. (S13) to the force-dependent factor by Eqs. (5) and (6) in the text.

The detailed balance condition in equilibrium for the eight-state model provided by Eq. (S10)

In graph theory, a fundamental cycle basis of an undirected graph is a set of simple cycles that forms a basis of the cycle space of the graph. The number of fundamental cycles $N_{\rm fc}$ in a given connected graph is provided as $N_{\rm e}$–$N_{\rm v}$+1 where $N_{\rm e}$ is the number of edges and $N_{\rm v}$ is the number of vertices [5]. In the case of the eight-state chemomechanical network model [Figs. 1 (c)], the number of the fundamental cycles is obtained as $N_{\rm fc}$ = 9 because of $N_{\rm e}$=16 and $N_{\rm v}$=8. Therefore, the detailed balance conditions are uniquely provided by nine independent equations based on the fundamental cycles. The nine fundamental cycles are displayed in Fig. S1. Each fundamental cycle provides the following detailed balance conditions:

(1) Cycle <125471>

$$\hat{k}_{12}\omega_{25}^0 k_{54}\hat{k}_{47}\hat{k}_{71} \big/ k_{21}k_{17}\hat{k}_{74}\hat{k}_{45}\omega_{52}^0 = 1 \,. \qquad \text{(S14)}$$

(2) Cycle <56125>

$$k_{56}k_{61}\hat{k}_{12}\omega_{25}^0 \big/ \hat{k}_{65}\omega_{52}^0 k_{21}\hat{k}_{16} = K_{eq} \,. \qquad \text{(S15)}$$

(3) Cycle <347123>

$$k_{34}k_{47}\hat{k}_{71}\hat{k}_{12}k_{23} \big/ \hat{k}_{43}\hat{k}_{32}k_{21}k_{17}\hat{k}_{74} = K_{eq} \,. \qquad \text{(S16)}$$

(4) Cycle <1471>

$$\omega_{14}^0 k_{47}\hat{k}_{71} \big/ k_{41}^0 k_{17}\hat{k}_{74} = 1 \,. \qquad \text{(S17)}$$

(5) Cycle <8258>

$$k_{82}\omega_{25}^0\hat{k}_{58} \big/ \hat{k}_{28}k_{85}\omega_{52}^0 = 1 \,. \qquad \text{(S18)}$$

(6) Cycle <8568>

$$k_{85}k_{56}\hat{k}_{68} \big/ \hat{k}_{58}k_{86}\hat{k}_{65} = K_{eq} \,. \qquad \text{(S19)}$$

(7) Cycle <8238>

$$k_{82}k_{23}\hat{k}_{38}\big/\hat{k}_{28}k_{83}\hat{k}_{32} = K_{eq}\,. \tag{S20}$$

(8) Cycle <3473>

$$k_{34}k_{47}\hat{k}_{73}\big/\hat{k}_{43}k_{37}\hat{k}_{74} = K_{eq}\,. \tag{S21}$$

(9) Cycle <6176>

$$k_{61}k_{17}\hat{k}_{76}\big/\hat{k}_{16}k_{67}\hat{k}_{71} = K_{eq}\,. \tag{S22}$$

We can use these nine equations to reduce the number of unknown transition rates.

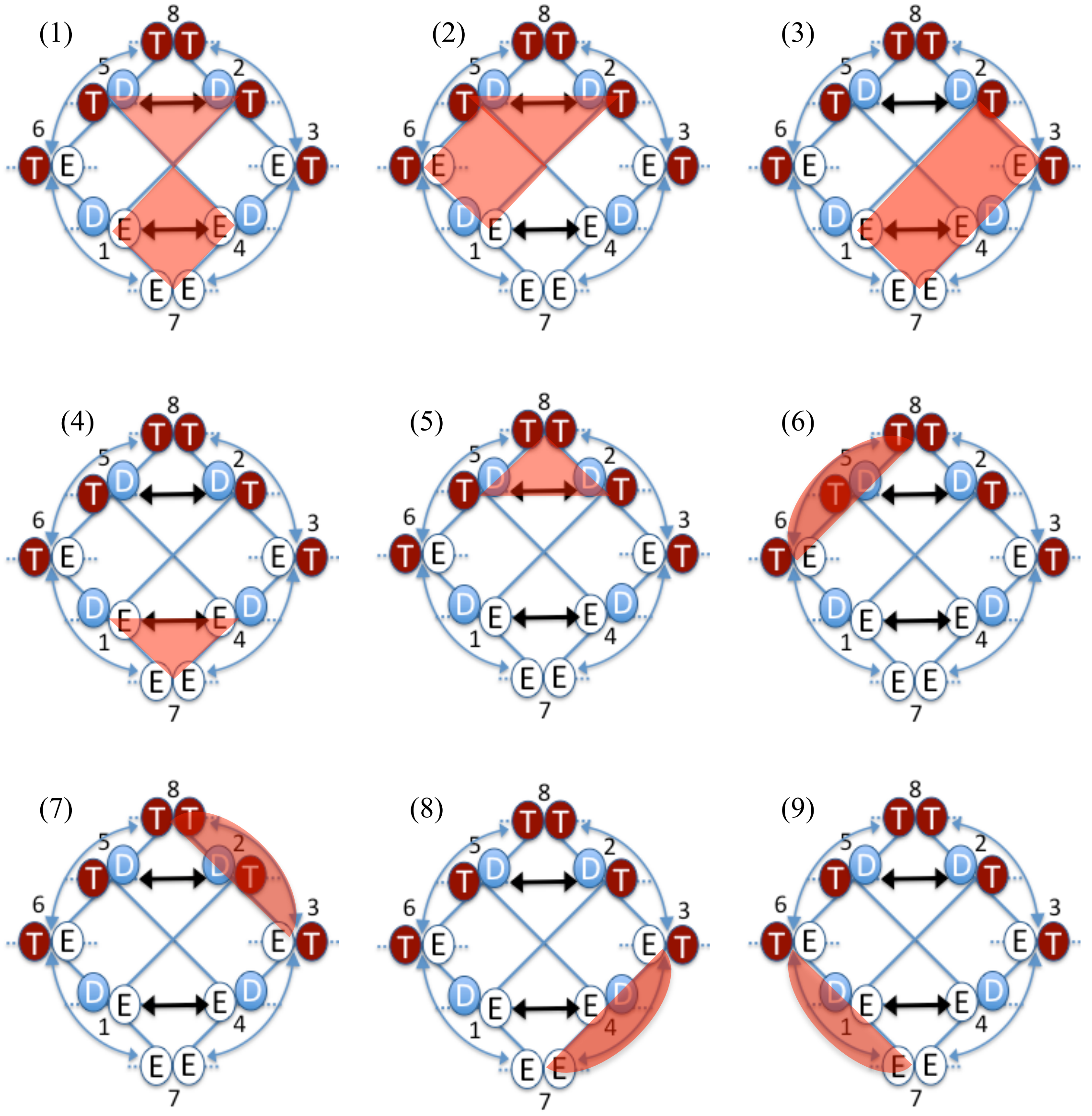

Figure S1. Nine fundamental cycles for the eight-state network model. The red mesh in each figure indicates a fundamental cycle.

Asymmetries and similarities between transition rates

There are 32 transitions among 8 states in the eight-state network model. On the basis of the intramolecular strain between two heads, we introduce asymmetries and similarities between the transition rates to reduce the number of independent parameters in the transition rates systematically. For example, we focus on the chemical transitions from state 8 (TT) to state 5 (TD) and from state 8 (TT) to state 2 (DT). Although both the transitions are ATP hydrolysis reactions, the one from state 8 to state 5 is ATP hydrolysis on the leading head that is strongly pulled backward, while the one from state 8 to state 2 is ATP hydrolysis on the trailing head that is strongly pulled forward. Therefore, the rates for these transitions would be affected by the intramolecular strain and thus should be different each other. This is an asymmetry between the chemical transitions due to the intramolecular strain. In the same way, for instance, we can also find a similarity between the transition from state 8 (TT) to state 2 (DT) and the transition from state 6 (TE) to state 1 (DE). Here, these transitions are ATP hydrolysis on the trailing heads that are strongly pulled forward because the leading heads at both states 8 (TT) and 6 (TE) are strongly bound to MT. In the same manner as the similarity, we also find a similarity between the transition from state 8 (TT) to state 5 (TD) and the transition from state 3 (ET) to state 4 (ED). According to the intramolecular strain between two heads that depends on the binding affinity of the partner head, we assume the following asymmetries and similarities between the transition rates:

(1) ATP hydrolysis/synthesis on the trailing head

$$\omega_{82} = \omega_{61}, \tag{S23a}$$

$$\omega_{28} = \omega_{16}. \tag{S23b}$$

(2) ATP hydrolysis/synthesis on the leading head

$$\omega_{85} = \omega_{34}, \tag{S24a}$$

$$\omega_{58} = \omega_{43}. \tag{S24b}$$

(3) ATP binding to/release from the trailing head

$$\omega_{38} = \omega_{76}, \tag{S25a}$$

$$\omega_{83} = \omega_{67}. \tag{S25b}$$

(4) ATP binding to/release from the leading head

$$\omega_{68} = \omega_{73}, \tag{S26a}$$

$$\omega_{86} = \omega_{37}. \tag{S26b}$$

(5) ADP binding to/release from the trailing head

$$\omega_{32} = \omega_{71}, \tag{S27a}$$

$$\omega_{23} = \omega_{17}. \tag{S27b}$$

(6) ADP binding to/release from the leading head

$$\omega_{65} = \omega_{74}, \tag{S28a}$$

$$\omega_{56} = \omega_{47} \,. \tag{S28b}$$

These similarity relations between the chemical transitions reduce the number of independent unknown transition rates from 32 to 20. If we substitute these 12 relations to Eqs. (S14)–(S22) that are provided by the detailed balance condition in equilibrium, we finally obtain the following 6 independent detailed balance conditions:

(1) Cycle <125471>

$$k_{54} = \hat{k}_{45} k_{17} k_{61} \Big/ \left(\hat{k}_{16} \hat{k}_{71} K_{eq} \right) . \tag{S29}$$

(2) Cycle <56125>

$$k_{21} = k_{47} \hat{k}_{12} k_{61} \omega^0_{25} \Big/ \left(\hat{k}_{74} \omega^0_{52} \hat{k}_{16} K_{eq} \right) . \tag{S30}$$

(4) Cycle <1471>

$$\omega^0_{41} = \omega^0_{14} \hat{k}_{71} k_{47} \Big/ \left(\hat{k}_{74} k_{17} \right) . \tag{S31}$$

(5) Cycle <8258>

$$\hat{k}_{43} = \omega^0_{52} \hat{k}_{16} k_{34} \Big/ \left(k_{61} \omega^0_{25} \right) . \tag{S32}$$

(6) Cycle <8568>

$$k_{86} = \hat{k}_{68} k_{47} k_{61} \omega^0_{25} \Big/ \left(\hat{k}_{74} \omega^0_{52} \hat{k}_{16} K_{eq} \right) . \tag{S33}$$

(7) Cycle <8238>

$$k_{83} = k_{61} k_{17} \hat{k}_{38} \Big/ \left(\hat{k}_{16} \hat{k}_{71} K_{eq} \right) . \tag{S34}$$

Here, K_{eq} is the equilibrium constant,

$$K_{eq} = \left. \frac{[\mathrm{ADP}][\mathrm{P}]}{[\mathrm{ATP}]} \right|_{eq} \simeq 4.9 \times 10^{11} \mu\mathrm{M}^{6}, \tag{S32}$$

where $[\mathrm{ATP}]$, $[\mathrm{ADP}]$, and $[\mathrm{P}]$ are the concentrations of ATP, ADP, and Pi (inorganic phosphate), respectively. As a result, the number of independent unknown transition rates is reduced to 14 from 20. Furthermore, since the transition rates between states 1 (DE) and 2 (DT) are ATP binding to/release from the leading head and the transition rates between states 4 (ED) and 5 (TD) are ATP binding to/release from the trailing head, although these partner heads are weakly bound to MT, we can expect the following approximate relations:

(1) ATP binding to/release from the leading head:

$$\omega_{12} \approx \omega_{68} = \omega_{73} , \tag{S35a}$$

$$\omega_{21} \approx \omega_{86} = \omega_{37} \,. \tag{S35b}$$

(2) ATP binding to/release from the trailing head:

$$\omega_{45} \approx \omega_{38} = \omega_{76}, \tag{S36a}$$

$$\omega_{54} \approx \omega_{83} = \omega_{67}. \tag{S36b}$$

Unbinding of kinesin from microtubule

In the eight-state model, all the states at which one head is strongly bound to MT and the other head is weakly bound to MT, i.e., states 2 (DT), 5 (TD), 1 (DE), and 4 (ED), are assumed to yield the mechanical step transitions. The detachment of kinesin from MT would more frequently occur via the states related to the mechanical step transitions than the other states 8 (TT), 7 (EE), 6 (TE), and 3 (ET) at which both the heads are strongly bound to MT. We, therefore, introduce unbinding transitions from MT via those states related to the mechanical transitions. In the 8-state mode, a state (DD) where both the heads are weakly bound to MT is omitted, although it is expected to most frequently lead the detachment of kinesin from MT [3]. However, we can approximately take into account the contribution from the unbinding via the state (DD) by introducing effective rates for the unbinding processes via states 2 (DT), 5 (TD), 1 (DE), and 4 (ED), since these states are directly connected to the state (DD) [Figs 1]. We introduce the following effective rates for the unbinding transitions via states 2, 5, 1, and 4 into an unbinding/absorbing state "0" under the backward and forward loads, respectively:

$$\omega_{i0}^{b} = k_{i0}^{b}\Omega_{i0}^{b}(F), \tag{S37a}$$

$$\omega_{i0}^{f} = k_{i0}^{f}\Omega_{i0}^{f}(F), \tag{S37b}$$

where k_{i0}^{b} and k_{i0}^{f} are zero-force unbinding rates for the backward and forward loads and $\Omega_{i0}^{b}(F)$ and $\Omega_{i0}^{f}(F)$ are force-dependent factors for the unbinding transition under the backward and forward loads,

$$\Omega_{i0}^{b}(F) = \exp\left[|F|/F_{i0}^{b}\right]. \tag{S38a}$$

$$\Omega_{i0}^{f}(F) = \exp\left[|F|/F_{i0}^{f}\right]. \tag{S38b}$$

Here, F_{i0}^{b} and F_{i0}^{f} are the force scales with respect to the force dependence of the unbinding transition from the state i under the backward and forward loads, respectively.

According to Hill's method for the mean time to absorption [7,8], the mean time of binding or unbinding rate can be calculated using a modified diagram in which each absorption state is replaced by a one-way cycle back to a starting state. In this study, we examined states 5 (TD) and 2 (TD) as the starting state to determine the mean time of binding and then obtained almost same unbinding properties of kinesin at low backward loads, while we found that the initial condition mainly affects force dependence of the unbinding rate at high forces around the stall force. All the results shown in this study are calculated using state 2 (DT) as the starting state.

Transition rates determined by fitting to available experimental data of kinesin's motor properties

Table S1. The chemical transition rates in the eight-state network model.
The definition of the parameters is provided at "Motor dynamics" in the Method section.

Parameters	Meaning	Values
$k_{82} = k_{61}$	ATP hydrolysis on the trailing head	150 s^{-1}
$\hat{k}_{28} = \hat{k}_{16}$	ATP synthesis on the trailing head	2.2x10^{-7}(μMs)$^{-1}$
$k_{85} = k_{34}$	ATP hydrolysis on the leading head	10 s^{-1}
$\hat{k}_{58} = \hat{k}_{43}$	ATP synthesis on the leading head *	3.9x10^{-11}(μMs)$^{-1}$
$k_{38} = k_{76}$	ATP binding to the trailing head	6.00 (μMs)$^{-1}$
$k_{83} = k_{67}$	ATP release from the trailing head *	0.464 s^{-1}
$k_{68} = k_{73}$	ATP binding to the leading head	4.00 (μMs)$^{-1}$
$k_{86} = k_{37}$	ATP release from the leading head *	104 s^{-1}
$\hat{k}_{45}$	ATP binding to the trailing head	4.00 (μMs)$^{-1}$
k_{54}	ATP release from the trailing head *	0.309 s^{-1}
$\hat{k}_{12}$	ATP binding to the leading head	4.00 (μMs)$^{-1}$
k_{21}	ATP release from the leading head *	104 s^{-1}
$k_{32} = k_{71}$	ADP binding to the trailing head	0.900 (μMs)$^{-1}$
$k_{23} = k_{17}$	ADP release from the trailing head	50.0 s^{-1}
$k_{65} = k_{74}$	ADP binding to the leading head	11.0 (μMs)$^{-1}$
$k_{56} = k_{47}$	ADP release from the leading head	550 s^{-1}

* Determined by the steady state balance condition.

Table S2. The mechanical step transition rates in the eight-state network model.

The definition of the parameters is provided at "Motor dynamics" in the Method section.

Parameters	Meaning	Values
ω_{25}^{0}	Forward transition from state 2 to state 5	3000 s^{-1}
ω_{52}^{0}	Backward transition from state 5 to state 2	8.00 s^{-1}
F_{25}	Force dependence on the 2–5 transition	0.747 pN
θ_{25}	Load distribution between states 2 and 5	0.75
ω_{14}^{0}	Forward transition from state 1 to state 4	15.0 s^{-1}
ω_{41}^{0}	Backward transition from state 4 to state 1*	13.5 s^{-1}
F_{14}	Force dependence on the 1–4 transition	0.747 pN
θ_{14}	Load distribution between states 1 and 4	0.999

* Determined by the extended steady state balance condition.

Table S3. The unbinding transition rates in the eight-state network model.

The definition of the parameters is provided at "Motor dynamics" in the Method section.

Parameters	Meaning	Values
k_{20}^{b}	Unbinding from state 2 (backward load)	2.00 s^{-1}
k_{20}^{f}	Unbinding from state 2 (forward load)	60.0 s^{-1}
F_{20}^{b}	Force dependence on the 2–0 unbinding (backward load)	7.47 pN
F_{20}^{f}	Force dependence on the 2–0 unbinding (forward load)	0.979 pN
k_{50}^{b}	Unbinding from state 5 (backward load)	3.00 s^{-1}
k_{50}^{f}	Unbinding from state 5 (forward load)	18.0 s^{-1}
F_{50}^{b}	Force dependence on the 5–0 unbinding (backward load)	2.74 pN
F_{50}^{f}	Force dependence on the 5–0 unbinding (forward load)	14.7 pN
k_{10}^{b}	Unbinding from state 1 (backward load)	0.200 s^{-1}
k_{10}^{f}	Unbinding from state 1 (forward load)	60.0 s^{-1}
F_{10}^{b}	Force dependence on the 1–0 unbinding (backward load)	7.47 pN
F_{10}^{f}	Force dependence on the 1–0 unbinding (forward load)	0.979 pN
k_{40}^{b}	Unbinding from state 4 (backward load)	2.00 s^{-1}
k_{40}^{f}	Unbinding from state 4 (forward load)	18.0 s^{-1}
F_{40}^{b}	Force dependence on the 4–0 unbinding (backward load)	2.74 pN
F_{40}^{f}	Force dependence on the 4–0 unbinding (forward load)	14.7 pN

On the basis of the binding affinity of the leading and trailing heads for MT at each state, we approximately assume that F_{50}^{b} and F_{50}^{f} would be equal to F_{40}^{b} and F_{40}^{f}, respectively, and F_{20}^{b} and F_{20}^{f} would be equal to F_{10}^{b} and F_{10}^{f}, respectively [Table S3]. We expected that F_{40}^{b} and F_{50}^{b} would be smaller than F_{40}^{f} and F_{50}^{f} so that states 4 (ED) and 5 (TD) would be sensitive to backward load because the leading head at these states is weakly bound to MT. In the same way, we expected that F_{10}^{f} and F_{20}^{f} would be smaller than F_{10}^{b} and F_{20}^{b} so that states 1 (DE) and 2 (DT) would be sensitive to forward load because the trailing head at these states is weakly bound to MT. We also assumed that the unbinding rates for forward load, k_{10}^{f}, k_{20}^{f}, k_{40}^{f}, and k_{50}^{f} are basically larger than that for backward load, k_{10}^{b}, k_{20}^{b}, k_{40}^{b}, and k_{50}^{b}, respectively. This is qualitatively consistent with the direction dependence of applied load on unbinding force distribution of kinesin's single head from MT [9].

Backward velocity under superstall load

The eight-state model slightly overestimates the absolute value of the backward velocity under superstall load at the high ATP concentration of 1 mM, while the increase in the backward velocity with increasing ATP concentration, which has been shown by experiment,[10] can be described by the eight-state model.

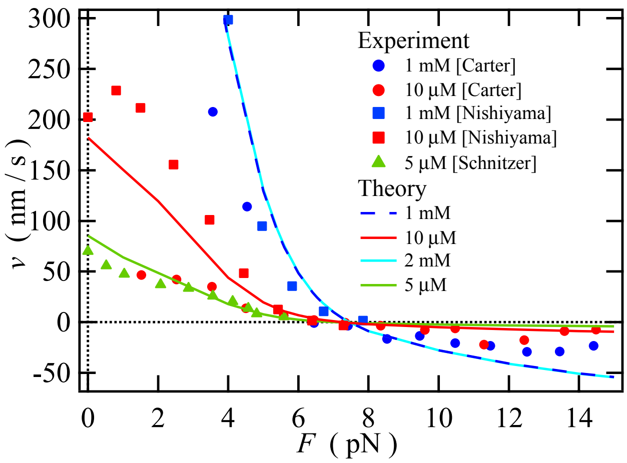

Figure S2. Backward load dependence of the motor velocity at several ATP concentrations.

Efficiencies of the molecular motor kinesin

In the eight-state model, the motor velocity is given by nonequilibrium steady state excess fluxes ΔJ_{ij}^{st} for the mechanical step transitions as follows:

$$v = l\left[\Delta J_{25}^{st} + \Delta J_{14}^{st}\right], \qquad \text{(S39)}$$

where l is the step length (8 nm) and ΔJ_{ij}^{st} is given by Eq. (2) in the steady state. In the same way, the ATP hydrolysis rate that corresponds to the average number of ATP hydrolysis per second is given by

$$\Delta J(\text{hydrolysis}) = \Delta J_{82}^{st} + \Delta J_{61}^{st} + \Delta J_{85}^{st} + \Delta J_{34}^{st}\,. \quad \text{(S40)}$$

A chemomechanical transduction efficiency η of a molecular motor that moves with velocity v against a load force F powered by ATP hydrolysis with the rate $\Delta J(\text{hydrolysis})$ is given by[4,11]

$$\eta = Fv / \left[\Delta\mu \Delta J(\text{hydorlysis}) \right]. \quad \text{(S41)}$$

Figure S3 (a) shows the chemomechanical transduction efficiency η as a function of external load at several ATP concentrations under constant ADP and Pi concentration of 5 μM. The maximum efficiency at the saturated ATP concentration ($\Delta\mu = 30.6 k_{\mathrm{B}} T$) is lower than that provided by Lipelt and Lipowsky and its load dependence at high load is slightly different from their results[4]. The loss in the transduction of the free-energy input $\Delta\mu$ provided by ATP into the mechanical work should be attributable to heat that irreversibly dissipates through the viscous friction of the probe [12] and that irreversibly dissipates from the motor due to chemical and mechanical slip cycles.

In Fig. S3 (b), the ratio of the number of forward step to the number of ATP hydrolysis, i.e., the chemomechanical coupling efficiency, is displayed as a function of the external load. At the saturated ATP concentration of 1 mM, the ratio of about 0.93 without the external load indicating a tight coupling between ATP hydrolysis and the mechanical forward step is in agreement with experimental observations [13-15]. From Fig. S3 (b), we also find that the ratio gradually decreases as the ATP concentration decreases. However, there is not enough experimental information to determine whether or not the tight coupling is still kept even at the low ATP concentrations. If the strong coupling comparable with the saturated ATP level is achieved by kinesin even at the low ATP concentrations, the eight-state model implies a possibility that the mechanical transition from state 1 to state 4 would also be a force-generating power stroke transition but the asymmetry of the transition between states 1 and 4 would not be so large compared with the transition between states 2 and 5. In this case, the thermodynamic equilibrium condition at the infinitely diluted concentration of ATP should be satisfied by the steady state balance condition [1,3].

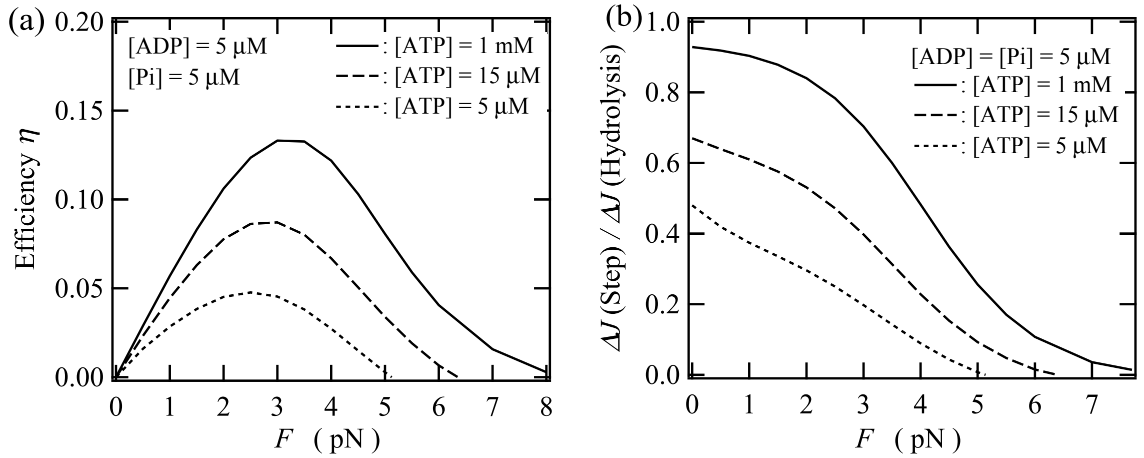

Figure S3. Load dependences of (a) a chemomechanical transduction efficiency $\eta = Fv / \left[\Delta\mu \Delta J \left(\text{hydorlysis} \right) \right]$ and (b) the ratio of the number of forward steps to the number of ATP hydrolysis $\Delta J\left(\text{step}\right) / \Delta J\left(\text{hydrolysis}\right)$, i.e., a chemomechanical coupling efficiency, where $\Delta\mu$ is a chemical potential difference given by $\Delta\mu = k_{\mathrm{B}} T \ln\left\{\left(K_{eq}\left[\text{ATP}\right]\right) / \left(\left[\text{ADP}\right]\left[\text{Pi}\right]\right)\right\}$ with the equilibrium constant K_{eq}= 4.9 x 10^{11} μM [6]; $\Delta J\left(\text{hydrolysis}\right)$, the total excess fluxes of ATP hydrolysis; $\Delta J\left(\text{step}\right)$, the total excess fluxes related to the mechanical transitions.

References

1. Liepelt, S. & Lipowsky, R. Steady-state balance conditions for molecular motor cycles and stochastic nonequilibrium processes. *Europhys. Lett.* **77,** 50002 (2007).
2. Seifert, U. Stochastic thermodynamics of single enzymes and molecular motors. *Eur Phys J E Soft Matter* **34,** 1–11 (2011).
3. Liepelt, S. & Lipowsky, R. Kinesin's Network of Chemomechanical Motor Cycles. *Phys. Rev. Lett.* **98,** 258102 (2007).
4. Liepelt, S. & Lipowsky, R. Impact of Slip Cycles on the Operation Modes and Efficiency of Molecular Motors. *J Stat Phys* **141,** 1–16 (2010).
5. Wallis, W. D. *A Beginner's Guide to Graph Theory*. (Springer Science & Business Media, 2010). doi:10.1007/978-0-8176-4580-9
6. Schief, W. R., Clark, R. H., Crevenna, A. H. & Howard, J. Inhibition of kinesin motility by ADP and phosphate supports a hand-over-hand mechanism. *Proc. Natl. Acad. Sci. U.S.A.* **101,** 1183–1188 (2004).
7. Hill, T. L. Interrelations between random walks on diagrams (graphs) with and without cycles. *Proc. Natl. Acad. Sci. U.S.A.* **85,** 2879–2883 (1988).
8. Hill, T. L. *Free energy transduction and biochemical cycle kinetics*. (Springer, 1989).

9. Uemura, S. & Ishiwata, S. Loading direction regulates the affinity of ADP for kinesin. *Nat Struct Biol* **10,** 308–311 (2003).
10. Carter, N. J. & Cross, R. A. Mechanics of the kinesin step. *Nature* **435,** 308–312 (2005).
11. Liepelt, S. & Lipowsky, R. Operation modes of the molecular motor kinesin. *Phys. Rev. E* **79,** 011917 (2009).
12. Toyabe, S. & Muneyuki, E. Single molecule thermodynamics of ATP synthesis by F1-ATPase. *New J. Phys.* **17,** 015008 (2015).
13. Hua, W., Young, E. C., Fleming, M. L. & Gelles, J. Coupling of kinesin steps to ATP hydrolysis. *Nature* **388,** 390–393 (1997).
14. Schnitzer, M. J. & Block, S. M. Kinesin hydrolyses one ATP per 8-nm step. *Nature* **388,** 386–390 (1997).
15. Coy, D. L., Wagenbach, M. & Howard, J. Kinesin takes one 8-nm step for each ATP that it hydrolyzes. *J. Biol. Chem.* **274,** 3667–3671 (1999).
